# Adaptation of a Bacterial Bioluminescent Assay to Monitor Bioeffects of Gold Nanoparticles

**DOI:** 10.3390/bioengineering9020061

**Published:** 2022-02-03

**Authors:** Moustafa R. Yehia, Tatyana E. Smolyarova, Alexandr V. Shabanov, Ekaterina S. Sushko, Gennady A. Badun, Nadezhda S. Kudryasheva

**Affiliations:** 1 Biophysics Department, Siberian Federal University, 660041 Krasnoyarsk, Russia; n-qdr@yandex.ru; 2 Institute of Physics SB RAS, Federal Research Center ‘Krasnoyarsk Science Center SB RAS’, 660036 Krasnoyarsk, Russia; smol.nano@yandex.ru (T.E.S.); alexch_syb@mail.ru (A.V.S.); kkovel@yandex.ru (E.S.S.); 3 Institute of Biophysics SB RAS, Federal Research Center ‘Krasnoyarsk Science Center SB RAS’, 660036 Krasnoyarsk, Russia; 4 Department of Chemistry, Moscow State University, 119991 Moscow, Russia; badunga@yandex.ru

**Keywords:** gold nanoparticles, luminous marine bacteria, bioassay, hormesis, tritium, reactive oxygen species

## Abstract

Our current study aimed to adapt a bioluminescent bacteria-based bioassay to monitor the bioeffects of gold nanoparticles (AuNPs). Luminous marine bacteria *Photobacterium phosphoreum* and AuNPs modified with polyvinylpyrrolidone were employed; low-concentration (≤10^−3^ g/L) bioeffects of AuNPs were studied. Bioluminescence intensity was used as an indicator of physiological activity in bacteria. Two additional methods were used: reactive oxygen species (ROS) content was estimated with a chemiluminescent luminol method, and bacterial size was monitored using electron microscopy. The bacterial bioluminescent response to AuNPs corresponded to the “hormesis” model and involved time-dependent bioluminescence activation, as well as a pronounced increase in the number of enlarged bacteria. We found negative correlations between the time courses of bioluminescence and the ROS content in bacterial suspensions, demonstrating the relationship between bioluminescence activation and bacterial ROS consumption. The combined effects of AuNPs and a beta-emitting radionuclide, tritium, revealed suppression of bacterial bioluminescent activity (as compared to their individual effects) and a reduced percentage of enlarged bacteria. Therefore, we demonstrated that our bacteria-based bioluminescence assay is an appropriate tool to study the bioeffects of AuNPs; the bioeffects can be further classified within a unified framework for rapid bioassessment.

## 1. Introduction

It is recognized that materials at the nanoscale possess unique optical, magnetic, catalytic, and electronic properties that differ from those of their bulk form [1,2,3,4]. These properties can be exploited to tackle a host of industrial, medical, and research problems. Modern developments in facile and high-yield methods have provided the impetus for an astounding increase in biological applications of nanoparticles (NPs) [5,6,7,8]. Whilst a variety of metals, such as silver and copper, are employed in industrial NP applications, gold is clinically utilized for its inert nature, biocompatibility, and propensity for biomolecular ligand immobilization [9,10,11,12]. These properties diversify the applicability of gold NPs to include radioprotection, bioimaging, biosensing, and drug delivery. However, this increased use of NPs has been accompanied by inconsistent toxicological studies. An overarching problem in NP toxicity assessment is the variability in protocols used to determine toxicity. These protocols utilize various molecular and chemical techniques, cell lines, and experimental conditions, which result in incomparable toxicity conclusions [13,14,15,16,17]. Consequently, it is essential to establish an informative, consistent, and unifying platform for the rapid assessment of nanomaterial effects on biological entities. 

An emerging candidate for this unification is a luminous bacterial bioassay. This assay has been extensively employed to monitor sample toxicity in complex media by considering bioluminescence as a physiological testing parameter [18,19,20,21,22,23]; it enjoys several advantages that include but are not limited to assay simplicity, high sensitivity, high-throughput capacity, and availability of devices for bioluminescence registration. Such advantages allow for large sample analysis and permit in-depth statistical processing, which are essential for investigating low-dose bioeffects of a stochastic nature [23,24,25,26]. Bioluminescent assays can operate at several levels of biological organization, either cellular or enzymatic. A classic example is the bioluminescent cellular method based on luminous marine bacteria that has been widely applied since the 1960s [27,28,29,30,31]. The enzymatic bioassay variant was first proposed in 1990 for ecotoxicological monitoring [31]; extensive utilization since the 1980s has highlighted its numerous advantages [32,33,34,35]. The primary physicochemical bioluminescent mechanisms and the nature of their interactions in bioluminescent systems have been extensively discussed [36,37,38,39]. In our previous works, we exploited the characteristics of bacterial and enzymatic bioluminescent bioassays to investigate the low-dose effects of alpha [40,41,42], beta [43,44], and gamma radiation [24]. The radioprotective properties of humic substances, products of natural oxidative decomposition of organic compounds in soils, were also demonstrated in solutions of alpha- and beta-emitting radionuclides [45,46,47,48]. Our previous works have revealed that bacterial responses to low-dose exogenous compounds correspond to the “hormesis” model [49,50]. This biological response is characterized by three main stages: stress recognition, stimulation, and subsequent inhibition of organism vital functions, i.e., toxicity [51,52,53,54].

Recently, we began to use bioluminescence assays to evaluate the bioeffects of nanostructures of natural and artificial origin. We studied NPs with varied core material and surface modification [55,56,57,58]. Fullerene derivatives with diverse carbon cage sizes and oxygen substituent amounts, as well as core iron oxides, were under investigation. We compared characteristics of NP bioeffects, such as toxic and antioxidant/pro-oxidant properties. We demonstrated correlations between the characteristics and surface modification, e.g., amount of oxygen substituent in fullerene carbon cages, hydrophobicity of surface modifiers, as well as involvement of exo- or endohedral metal in the fullerene structure. Moreover, we highlighted the involvement of the conjugated π-system of the carbon cage in fullerenol bioeffects [55]. We further demonstrated an active role of reactive oxygen species (ROS) in the bioeffects of fullerenol NPs [56]; ROS content was monitored using the chemiluminescence luminol method [59,60]. This technique determines integral ROS content by assuming a dynamic equilibrium of the different intra- and extracellular ROS. The luminol technique is also highly convenient when tandemly used with bioluminescence measurements, since both techniques use the same instrumentation for the registration of luminescence kinetics. Hence, our previous investigations provided a collection of data that forms a fundamental basis for analyzing and comparing the bioeffects of NPs of different structures (different cores and surface modifications) at cellular and enzymatic levels. We found correlations between structural characteristics and toxic and anti-oxidant effects, which can constitute a predictive tool for appropriate NP selection in pharmacological, biomedical, and ecological applications. 

Our current paper focuses on adapting the bacteria-based bioluminescent assay to investigate the bioeffects of gold nanoparticles (AuNPs). AuNPs have been extensively studied and are considered one of the most chemically neutral carriers of bioactive compounds and ligands in targeting local bioprocesses in organisms [61,62,63,64]. This study is necessary (1) to provide a consistent comparison of AuNP bioeffects with other nanoparticles and to compare these bioeffects with those of nanostructures previously studied using similar techniques and (2) to develop, through this, a classification of NP bioeffects based on structural characteristics, i.e., core type and surface modification. We plan to expand the number of classified NPs to include AuNPs with distinct surface modifications and AuNP nanocomposites. Changes in radiosensitivity of bacteria under exposure to AuNPs and the role of ROS in the molecular mechanisms of AuNP bioeffects are taken into special consideration.

Our study is aimed at:

(a) defining appropriate bioassay conditions (e.g., selection of bacterial growth stage) to study the bioeffects of AuNPs;
(b) investigating the peculiarities of low-concentration effects of AuNPs (10^−6^–10^−3^ g/L) on the bioluminescence of marine bacteria;
(c) revealing possible AuNP radioprotective effects in solutions of beta-emitting radionuclide tritium under conditions of low-dose irradiation (<0.1 Gy);
(d) evaluating the role of ROS in the bioeffects of AuNPs;
(e) elucidating correlations between bacterial cell morphology and bacterial bioluminescence responses in complex AuNP solutions.

AuNPs modified with polyvinylpyrrolidone, a widespread representative of biocompatible AuNPs, were chosen for our experiments. 

## 2. Materials and Methods

### 2.1. Preparations and Reagents

Hydrogen tetrachloroaurate(III) trihydrate (HAuCl_4_·3H_2_O) was procured from Alfa Aesar (ThermoFisher Inc., Karlsruhe, Germany). Luminol (C_8_H_7_N_3_O_2_), trisodium citrate (Na_3_C_6_H_5_O_7_), potassium ferricyanide (K_3_[Fe(CN)_6_]), and polyvinylpyrrolidone (PVP) were obtained from Sigma-Aldrich, St. Louis, MO, USA. Tryptone and yeast extract were obtained from Dia-M, Moscow, Russia. Sodium chloride (NaCl), magnesium chloride hexahydrate (MgCl_2_ 6H_2_O), calcium chloride (CaCl_2_), and potassium chloride (KCl) were obtained from Pancreac Applichem ITW Reagents, Germany. Bacterial agar was obtained from Difco Laboratories, Detroit, MI, USA. Potassium hydroxide (KOH) was obtained from Khimreactiv, Nizhny Novgorod, Russia. Tritiated water, HTO, JSC Isotope, Russia, was used as a source of tritium. HTO was added to NaCl solutions and mixed with the bacterial suspensions to the final specific radioactivities of 2, 10, 50, and 200 MBq/L. All the reagents were of analytical grade and used as received.

### 2.2. Bacterial Growth Conditions

Intact marine luminous bacterium, strain *Photobacterium phosphoreum* 1883 IBSO [65], was used as a bioassay to evaluate the effects of AuNPs on the bacterial cells. The strain was obtained from the Collection of Luminous Bacteria CCIBSO-863, Institute of Biophysics SB RAS, Krasnoyarsk, Russia. For cultivation of *P. phosphoreum*, a semisynthetic medium containing 10 g/L Tryptone, 28.5 g/L NaCl, 4.5 g/L MgCl_2_∙6H_2_O, 0.5 g/L CaCl_2_, 0.5 g/L KCl, 3 g/L yeast extract, and 12.5 g/L agar was used. 

*P. phosphoreum* was plated on 25 mL of semisynthetic agar and incubated at 25 °C for 17 and 24 h (exponential growth phase and stationary growth phase, respectively) in an incubator (WIS-20R, WiseCube Laboratory Instruments, Wertheim, Germany). Prior to experimentation, bacteria were collected by pipetting of 3% NaCl solution directly onto the agar to release bacteria. The bacterial suspension was then diluted to an optical density = 0.1 at 660 nm and stored at 4 °C for 30 min to allow bioluminescence stabilization prior to experimentation. The NaCl was of analytical grade. The 3% NaCl solutions were used to mimic a marine environment for the bacterial cells and to maintain osmotic processes. 

### 2.3. Preparation and Characterization of Gold Nanoparticles

AuNPs modified with PVP were prepared according to the sodium citrate reduction method [66]. Briefly, 5 mL of 0.1 mM sodium tricitrate solution was added to a 100 mL boiling solution of 0.5 mM HAuCl_4_, and the reaction was allowed to react for 30 min under continuous stirring. The sodium citrate nanoparticle solution was then allowed to stabilize for 24 h prior to ligand replacement. The resulting nanoparticle solution was resuspended in ethanol for ligand replacement. Subsequently, 5 mL of polyvinylpyrrolidone (PVP) aqueous solution (60 PVP molecules per nm^2^ of nanoparticle) was added to the sodium citrate nanoparticles and allowed to react overnight under continuous stirring. The final solution was centrifuged at 4000 RPM to remove impurities and unreacted reagents and redispersed in distilled water. Transmission electron microscopy (TEM) images and dynamic light scattering (DLS) confirmed the presence of spherical 15 nm AuNPs (Figure S1B,D in Supplementary Materials, respectively). Additionally, AuNP aggregate clusters appeared (post ligand replacement) and were also visualized microscopically (Figure S1A in Supplementary Materials). The size distribution of bare AuNPs (sharp peak at ~15 nm) and aggregated (broadened peak at ~80 nm) AuNPs in solution was further visualized by TEM size distribution (Figure S1C in Supplementary Materials). 

### 2.4. Luminescence Measurements

#### 2.4.1. Bioluminescence Assay Samples

Bioluminescence kinetics was studied in the bacterial suspensions of different compositions: bacteria (control samples); bacteria + AuNPs; bacteria + HTO; bacteria + HTO + AuNPs. The bacterial suspensions were prepared as follows. Control samples: 400 μL of non-radioactive bacteria suspensions was added to 1600 μL of 3% NaCl solution. Samples (bacteria + AuNPs): 400 μL of bacterial suspensions and 400 μL of AuNPs were added to 1200 μL of 3% NaCl solution. Radioactive samples (bacteria + HTO): 400 μL of bacterial suspensions was added to 50 μL of HTO in 1550 μL of 3% NaCl solution. Radioactive samples (bacteria + HTO + AuNPs): 400 μL of bacterial suspensions and 400 μL of AuNPs were added to 50 μL of HTO in 1150 μL of 3% NaCl solution.

#### 2.4.2. Bioluminescence Registration

To investigate the chronic effects of AuNPs on the bacterial cells, standard procedures for bioluminescence measurements were used [26,65]. Bioluminescence intensity time courses were measured for 21 h. The relative bioluminescence intensities, *I^rel^*, were calculated as ratios of the bioluminescence intensities of experimental and control suspensions. The time courses of *I^rel^* were studied at different concentrations of AuNPs and HTO and their combinations (see Section 2.4.1). The radioactive dose accumulated in the bacterial samples did not exceed 0.1 Gy. Optical density of AuNP solutions did not exceed 0.1 in maxima of the bioluminescence and chemiluminescence light emittance—490 and 425 nm, respectively; hence, the effect of “optic filter” was excluded [67]. All measurements were carried out in 5–10 replicates; error did not exceed 10%. 

#### 2.4.3. Chemiluminescence Assay for Evaluation of Reactive Oxygen Species Content

We used the luminol chemiluminescence method [59,60] to evaluate the content of reactive oxygen species (ROS) in the experimental bacterial suspensions. Chemiluminescence registration was carried out just after the bioluminescence measurements in the same bacterial samples. The calibration dependence was preliminarily determined as chemiluminescence intensity vs. Н_2_О_2_ concentration; Н_2_О_2_ was considered a representative of the ROS family. Concentrations of alkaline luminol and K_3_[Fe(CN)_6_] solutions were 5 × 10^−4^ М and 5 × 10^−3^ M, respectively. Calibration dependency is presented in Figure S2 in Supplementary Materials.

ROS content was evaluated in bacterial suspensions in 3% NaCl solutions at different concentrations of AuNPs (10^−6^–10^−3^ g/L), HTO (2, 10, 200 MBq/L) and their combinations at various times of exposure. Maximal chemiluminescence intensity was determined in bacterial suspensions after bioluminescence measurements. To provide this, the luminol solutions were added to the bacterial samples. Then, the chemiluminescence reaction was initiated by 75 μL solution of K_3_[Fe(CN)_6_] (pH 11.24) through the injection system. Chemiluminescence intensity was used to calculate ROS content in the experimental solutions via the calibration dependence (Figure S2 in Supplementary Materials). Relative values of ROS content, *ROS^rel^*, were calculated as ratios of ROS content in experimental and control solutions; kinetics of *ROS^rel^* were plotted. All measurements were carried out in 5 replicates; error did not exceed 10%. 

### 2.5. Microscopy

Samples for electron microscopy were prepared identically to those described in Section 2.4.1: control bacteria; bacteria + AuNPs; bacteria + HTO; bacteria + HTO + AuNPs. All samples were allowed to react for 3 h prior to fixation. Afterwards, the samples were initially mixed with 2% formaldehyde solution (1:1 *v*/*v*) for 1 h prior to deposition on the absorbing matrix and allowed to air dry for 15 min prior to imaging. Microscopy settings and sample preparation were replicated according to a method developed for biological object fixation on inverse opals, as previously described in [68]. Briefly, silica inverse opals were prepared by a sol-gel method to study biological objects. Submicron spherical particles were obtained from polymethylmethacrylate to produce 100 to 500 nm particles that were ordered into an inverse opal crystal matrix. This created an open system of pores up to 400 nm in size that was used for sample adsorption. 

### 2.6. Equipment

Time courses of luminescence intensity were registered by Luminoskan Ascent (Thermo Fisher 219 Corp., Waltham, MA, USA). Optical density and absorbance of the bacterial and nanoparticle solutions were measured by an UVIKON-943 double-beam spectrophotometer (KONTRON Instruments, Milano, Italy). All luminescence measurements were carried out at +20 °C. An SU3500 SEM (Hitachi, Tokyo, Japan) was used for bacterial morphological analysis. AuNP shape and size were determined by transmission electron microscopy (TEM) (Hitachi S-5500) and Zetasizer (Malvern instruments, Malvern, UK), respectively.

### 2.7. Statistical Processing

To evaluate correlations between time courses of bioluminescence intensity and ROS content (*I^rel^* and *ROS^rel^*, respectively), a statistical dependence between the rankings of the two variables was analyzed [69], and Spearman’s rank correlation coefficients (*r*) were calculated. The application of this method was justified with a moderate kit of data sets, as well as a lack of normal distribution of *I^rel^* and *ROS^rel^*. 

## 3. Results and Discussion

### 3.1. Bacterial Growth Phase Selection

**Figure 1:**
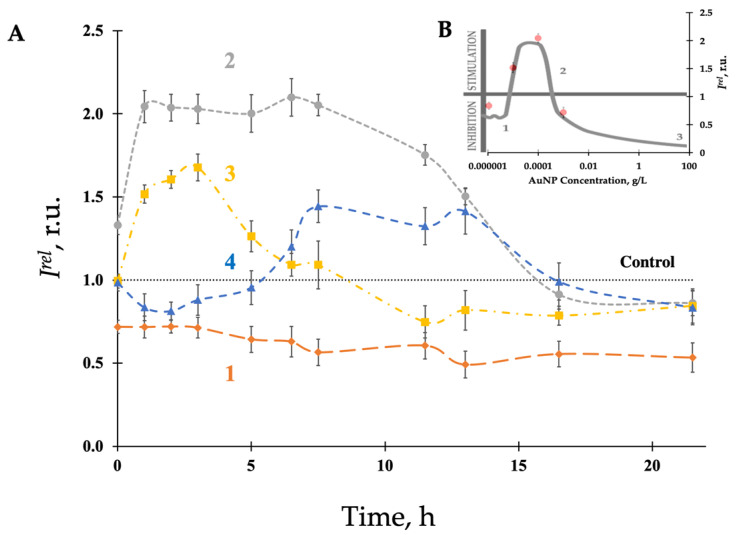
Bioluminescence intensity of bacteria, *I^rel^*, sampled at stationary growth phase (24 h). (**A**) Bioluminescence kinetics after exposure to AuNPs of different concentrations (color curves)**:** 10^−3^ g/L (**1**), 10^−4^ g/L (**2**), 10^−5^ g/L (**3**), and 10^−6^ g/L (**4**); (**B**) Bioluminescence intensity, *I^rel^*, at different concentrations of AuNPs (red points), 3-h exposure. Hormetic curve (gray color) is presented according to descriptions presented in [46]. Three stages of hormesis include: (1) stress recognition, (2) physiological activation, (3) inhibition of vital functions.

We chose spherical AuNPs (15 nm) functionalized with polyvinylpyrrolidone (PVP) as representatives of biocompatible nanoparticles. Our preliminary adaptation of the bioluminescence assay involved the choice of bacterial growth stage (exponential or stationary) for AuNP bioeffect assessment. Conventionally, most toxicity assays employing bacterial bioluminescence select bacteria from the exponential growth phase (17 h) due to maximal luminescence and sensitivity [70,71]. We sampled bacteria from both the exponential and stationary growth phases (17 and 24 h, respectively) and exposed them to four concentrations of AuNPs (10^−3^, 10^−4^, 10^−5^, 10^−6^ g/L). The bacteria sampled at the exponential stage of growth (17 h) did not show distinct differences at various concentrations of AuNPs (Figure S3 in Supplementary Materials). However, the bioluminescent kinetics of the bacteria sampled from the stationary growth phase (24 h) demonstrated considerable deviations from the control (Figure 1A), indicating suitability for studying AuNP bioeffects under low-concentration conditions. While the deviations (Figure 1A) may seem stochastic, they are particularly valuable when considered through the hormesis model, which supposes a nonlinear dependency of bioeffects on dosage (Figure 1B).

Figure 1B presents bioluminescence intensity, *I^rel^*, at four concentrations of AuNPs after 3 h exposure (red points). We observed a similar tendency at various other exposure times and AuNP concentrations. As seen from this figure, the nonlinear concentration dependence is consistent with the classic hormetic curve described in previous works on the hormesis phenomenon [72,73,74,75]. We can observe that AuNP concentrations approximately fall within the three classical stages of hormesis: stress recognition, physiological activation, and inhibition of vital functions. As a phenomenon, hormesis involves favorable biological responses to low exposures of stressors (e.g., chemical or radiation) [70]. In toxicology, hormesis is a dose-response phenomenon characterized by low-dose physiological stimulation and higher-dose inhibition, resulting in classical J-shaped or inverted U-shaped curves. This phenomenon is highly generalizable and independent of biological model, endpoint selection, stressor type, and biological level of organization (enzymatic, cellular, or whole organism). Nevertheless, although hormesis has been extensively studied in the past few decades, the hormesis mechanisms remain elusive. Therefore, experimental studies elucidating molecular mechanisms of hormesis responses, particularly in bacterial cells, are of significant interest. 

Hence, our current experiment revealed that the resultant bioeffect of AuNPs is concentration-dependent only for bacteria sampled at the stationary growth phase (24 h). We speculate that our results are due to differences in membrane composition between exponential and stationary phase bacteria. Given these results, we chose bacteria from the stationary growth phase for further experiments. 

### 3.2. Combined Effects of AuNPs and Tritium on Luminescent Bacteria

**Figure 2:**
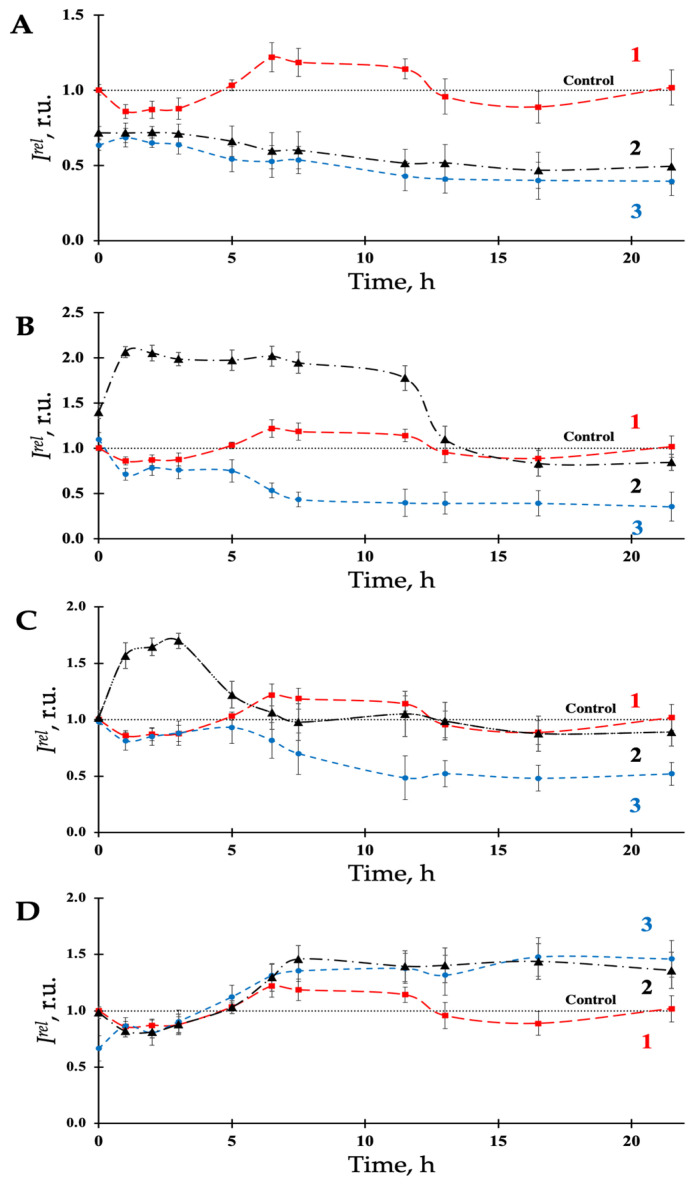
Bioluminescence intensity, *I^rel^*, in bacterial suspensions in the presence of HTO (2 MBq/L) and/or AuNPs of different concentrations (colored curves): (**1**) HTO, (**2**) AuNPs, AuNPs + HTO, (**3**) 2 MBq/L. Concentrations of AuNPs: (**A**)—10^−3^ g/L, (**B**)—10^−4^ g/L, (**C**)—10^−5^ g/L, (**D**)—10^−6^ g/L.3.3. Effects of AuNPs on Bacterial Bioluminescence and ROS Content.

To investigate the influence of low-intensity radioactive exposure, we used tritium, a low-energy (18.575 keV), beta-emitting radionuclide, applied as a component of tritiated water (HTO). The effects of tritium on luminous marine bacteria were previously studied in detail [24,26,38,43,44]. These studies showed that bioluminescence kinetic curves involved activation (*I^rel^* > 1) and inhibition (*I^rel^* < 1) stages and were described in terms of hormesis phenomena. Our current study similarly demonstrated a hormetic dependence with moderate bioluminescence inhibition and activation in response to HTO (2 MBq/L) exposure (Figure 2A–C, curve 1). 

Figure 2 highlights the individual and combined effects of HTO and AuNPs on bacterial luminescence. Several concentrations of AuNPs were applied (Figure 2A–D). We can observe that AuNPs and HTO independently produce bioluminescent activation (curves 1 and 2). However, combinations of AuNPs and HTO produce consistent bioluminescence inhibition (curve 3), excluding the lowest concentration of AuNPs (Figure 2D). Non-additivity of effects of AuNPs and HTO in complex solutions of AuNPs + HTO is apparent from the comparison of kinetic curves 1, 2, and 3 in Figure 2A–D. This may allude to complex interactions of the solution components, which plausibly involve intercomponent and bacteria-component interactions. Our results confirmed the radiosensitizing capacity of AuNPs towards bacterial cells in solutions of beta-emitting radionuclide tritium. In biomedical applications, radiosensitization is a physical or chemical method used to amplify the deleterious effects of radiation exposure [76]. The clinical use of materials with radiosensitizing properties has brought significant attention to metal-based nanoparticle radiosensitization [77,78]. Despite the radiosensitizing capacity of AuNPs having been demonstrated, the underlying mechanism remains significantly debated [79]. 

**Figure 3:**
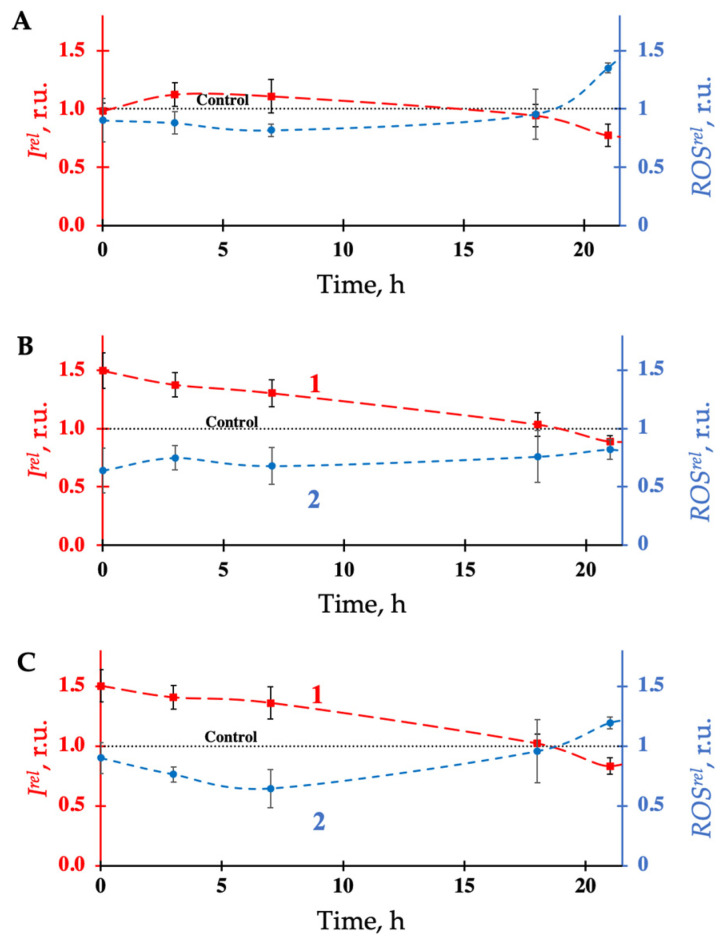
Kinetics of bacterial bioluminescent intensity, *I^rel^*, (**1**) (red color) and relative ROS content, *ROS^rel^*, (**2**) (blue color) in the presence of AuNPs of different concentrations: (**A**)—10^−3^ g/L; (**B**)—10^−4^ g/L; (**C**)—10^−5^ g/L. The ROS content in the control (non-radioactive) bacterial suspension decayed from 5.9 × 10^−6^М to 1.7 × 10^−6^ М during the duration of the experiments.

Physicochemical mechanisms of biological activation by biocompatible AuNPs are of particular interest. We studied the role of ROS in AuNP bioeffects on bacterial cells in additional bioluminescence and chemiluminescence experiments. The time courses of bioluminescence, *I^rel^*, (curve 1) and ROS content, *ROS^rel^*, (curve 2) in bacterial suspensions with different concentrations of AuNPs (10^−3^, 10^−4^, and 10^−5^ g/L) are presented in Figure 3A–C. A moderate increase in bioluminescence (*I^rel^* > 1) was observed with exposure to AuNPs at all three concentrations for most of the duration of bioluminescence monitoring (Figure 3A–C, curve 1). Inversely, corresponding decreases in ROS content (*ROS^rel^* < 1) were observed (curve 2). High negative correlation coefficients, r, between the time courses of *I^rel^* and *ROS^rel^* were found: A: –0.9; B: –0.7: C: –0.9, for Figure 3A–C, respectively (*p* < 0.05). These results corroborate our previous studies, wherein analogous negative correlations were found under exposure to radioactivity as a biological stressor [46]. We may conclude that low concentrations of AuNPs simultaneously intensify bioluminescence activation and ROS consumption. Biological regulation of ROS by AuNPs may involve the formation of weak hydroxyl bonds with adjacent water molecules, leading to lower ionization energy of surrounding media, as discussed in [80,81,82,83,84]. Further studies are required to elucidate the connection between ROS regulation and bioluminescence intensification.

### 3.3. Morphology of Bacterial Cells Exposed to AuNPs

**Figure 4:**
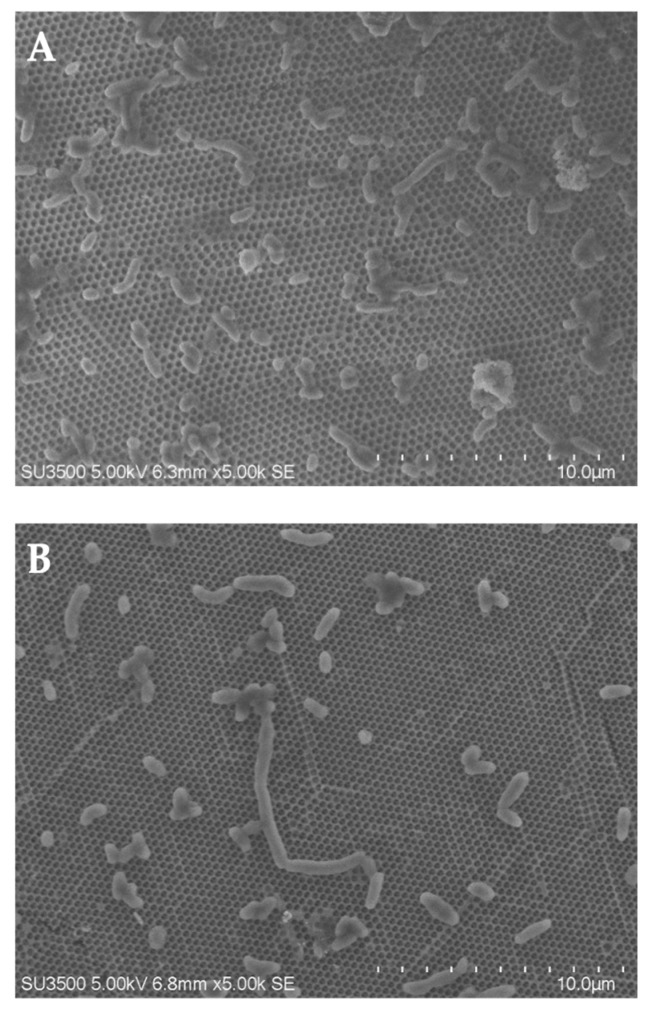
Scanning electron microscopy images of *P. phosphoreum* after 3 h. (**A**): control. (**B**): AuNPs (10^−3^ g/L).

We aimed to elucidate whether changes in cellular morphology correlated with bioluminescence activation or inhibition. Specifically, we utilized microscopy imaging to study bacterial cell morphology in four samples: (1) control bacteria; (2) bacteria + AuNPs; (3) bacteria + HTO; (4) bacteria + HTO + AuNPs:

(1) Control bacteria had average sizes between 0.5 and 2.5 μm, with uniform thickness and normally distributed sizes. Significantly large (>4 μm) and elongated bacteria constituted 7% of all cells.
(2) After exposing the bacteria to AuNPs, 10^−3^ g/L, we observed a change in the size distribution relative to the control; significantly large (>4 μm) and elongated bacteria constituted a larger percentage of the size distribution (27%) (Figure 4). The increase in bacterial size was accompanied by noticeable changes in bioluminescence intensity under AuNP exposure, as discussed previously (curve 1 in Figure 3A–C). Conceivably, exposure to AuNPs modified with PVP may have influenced bacterial growth cycles, as with other biocompatible surface ligands seen in [85]. This is supported by reports of biocompatible AuNPs intensifying cell division and increasing bacterial cell size in Gram-negative bacteria [86]. These results warrant further studies regarding the effects of AuNP exposure on bioluminescence dynamics and cellular morphology.
(3) Exposure to HTO, 50 MBq/L, revealed 16% significantly large (>4 μm) and elongated bacteria. Hence, we observed a moderate effect of HTO on cell length, particularly through abnormal elongation of cells. This moderate increase in bacterial size was accompanied by moderate bioluminescence activation under HTO exposure (curve 1, Figure 2A–D). We should note that the damaging effect of tritium (100 MBq/L) on the ultrastructure of lyophilized luminescent bacteria was observed previously [43]; however, the conditions of our current experiment did not reveal any structurally damaging effects, likely due to the use of intact bacteria and lower exposure times.
(4) Finally, we studied the combined effects of AuNPs and HTO, 10^−3^ g/L and 50 MBq/L, respectively. We observed a bacterial size distribution similar to HTO alone (15%). Nevertheless, unlike HTO alone, we observed abnormally elongated bacterial cells identical to those seen under AuNP exposure. Altogether, we observed a nonadditive inhibitory effect of HTO + AuNP on luminous marine bacteria, demonstrated in Figure 2A–C, (curves 3), accompanied by a decrease in the amount (up to 15%) of significantly large (>4 mm) and elongated bacteria, as compared to the effect of AuNPs alone (27%). These changes should be further investigated under the hypothesis of media ionization and intensification of membrane processes by HTO in bioluminescence activation [44].

## 4. Conclusions

At present, the biological effects of nanostructures are of significant interest to diverse fields of biomedicine and environmental technologies. The modifiability of nanoparticle surfaces diversifies their interactions with surrounding media and organisms. This complexity prevents the prediction of nanoparticle bioeffects based solely on physicochemical characteristics; integral and nonspecific biological assessment methods should be involved. Bioluminescent bioassays are prospective candidates for the comparison of the biological activity of various nanoparticles due to their simplicity and high-throughput capacity. 

In recent years, we have studied and compared the toxic and antioxidant properties of nanostructures with diverse cores (iron oxides and carbon) and ligands, as well as bioactive macromolecules of natural origin, i.e., humic substances. Our current study aimed to complement the series of studied nanostructures with AuNPs, which have been subject to extensive investigation and application, primarily due to their biologically inert nature. This study contributes to nanoparticle bioactivity classification per structural characteristics, which we aim to develop extensively. Based on recent advances in biogenic nanoparticle synthesis and characterization, we aim to utilize our method to further investigate the toxic profiles and applications of biogenic nanoparticles with varying core compositions and surface ligands [87,88,89,90,91]. We hope that the recent preponderance of data on biogenic nanoparticles can contribute to the elucidation of the mechanisms of nanoparticle toxicity and hormesis. 

Our current paper showed that luminous marine bacteria *P. phosphoreum* sampled at the stationary growth phase (in contrast to the exponential growth stage) are more sensitive to low-concentration suspensions of AuNPs (<10^−3^ g/L) and can hence be preferentially applied in a bioluminescent assay to monitor AuNP bioeffects. We chose AuNPs modified with polyvinylpyrrolidone due to their extensive use in research and industrial applications. The bacterial response to AuNP exposure corresponded to the “hormesis” model and involved dose-dependent activation and inhibition of bioluminescence, in addition to increased bacterial size. We found negative correlations between the time courses of bioluminescence and ROS content in bacterial suspensions, demonstrating that bacterial bioluminescence activation by AuNPs is concerned with ROS consumption by bacteria. The combined exposure to AuNPs and a beta-emitting radionuclide tritium revealed a suppression of bacterial bioluminescence (in contrast to the individual effects of AuNPs and tritium) and a reduced percentage of enlarged bacteria. Thus, we have demonstrated that our bacteria-based bioluminescence assay exhibits strong potential as an appropriate tool for studying and comparing the bioeffects of AuNPs and other bioactive nanocompounds. Additionally, we have demonstrated the potential of coupling our bioluminescent assay with other research methods that include microscopic and spectroscopic techniques to achieve a more nuanced understanding of nanomaterial toxicity profiles.

## Data Availability

Not applicable.

## Supplementary Materials

The following supporting information can be downloaded at: www.mdpi.com/article/10.3390/bioengineering9020061/s1, Figure S1: Characterization of AuNPs by transmission electron microscopy (TEM) and dynamic light scattering (DLS). Figure S2: Calibration curve for chemiluminescence luminol method for ROS evaluation. Dependence of chemiluminescence intensity on concentration of Н_2_О_2_. Figure S3: Bioluminescence kinetics of bacteria sampled at exponential phase of growth (17 h) in the presence of AuNPs.
