# Supplementary material for: Adaptation of a Bacterial Bioluminescent Assay to Monitor Bioeffects of Gold Nanoparticles
Source: Bioengineering (Basel). 2022 Feb 3;9(2):61. doi: 10.3390/bioengineering9020061 (PMC8868574; doi:10.3390/bioengineering9020061)
Supplement: Supplementary file 1 [file bioengineering-09-00061-s001.zip › bioengineering-1564113-SI.pdf]

Supplementary Materials

Adaptation of a Bacterial Bioluminescent Assay to Monitor Bioeffects of Gold Nanoparticles

Moustafa R. Yehia [1,*], Tatyana E. Smolyarova [2], Alexandr V. Shabanov [2], Ekaterina S. Sushko [2,3], Gennady A. Badun [4] and Nadezhda S. Kudryasheva [1,3]

[1] Biophysics Department, Siberian Federal University, 660041 Krasnoyarsk, Russia; n-qdr@yandex.ru
[2] Institute of Physics SB RAS, Federal Research Center 'Krasnoyarsk Science Center SB RAS', 660036 Krasnoyarsk, Russia; smol.nano@yandex.ru (T.E.S.); alexch_syb@mail.ru (A.V.S.); kkovel@yandex.ru (E.S.S)
[3] Institute of Biophysics SB RAS, Federal Research Center 'Krasnoyarsk Science Center SB RAS', 660036 Krasnoyarsk, Russia
[4] Department of Chemistry, Moscow State University, 119991 Moscow, Russia; badunga@yandex.ru
* Correspondence: moustafa.yehia@city.ac.uk

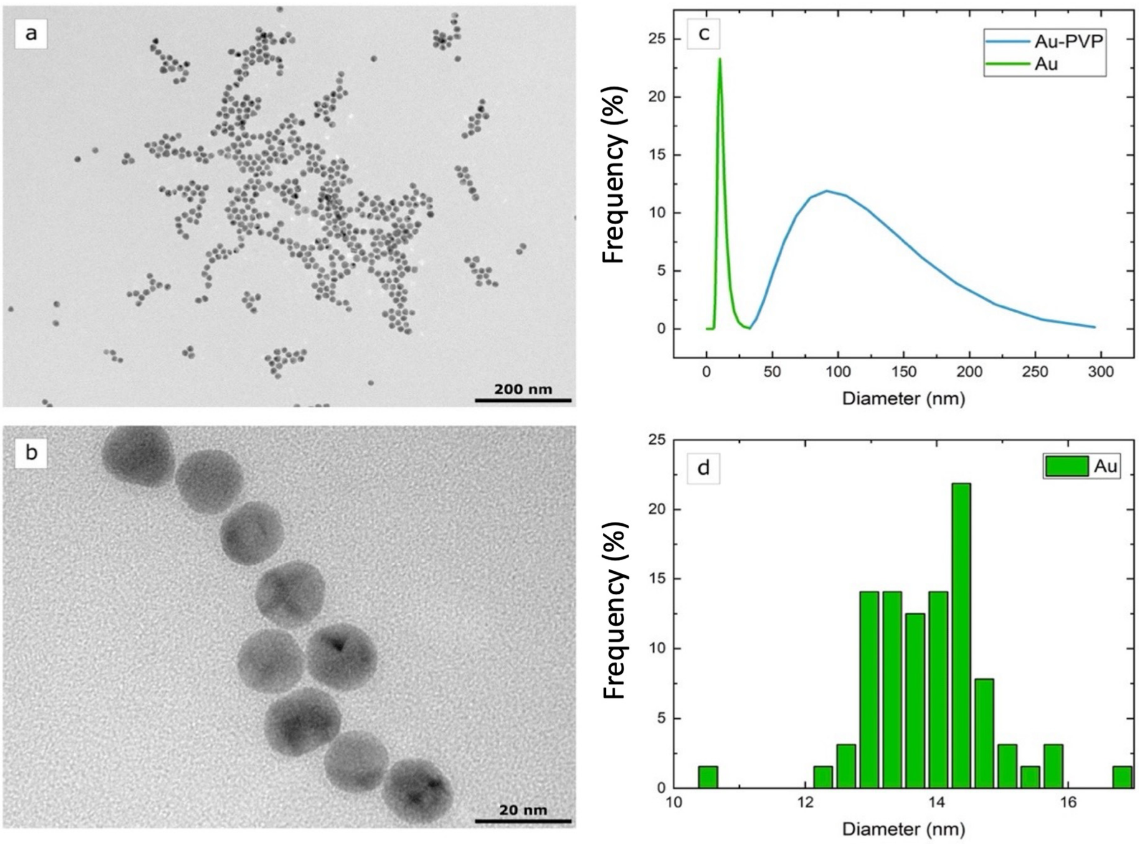

Figure S1. Characterization of AuNPs by transmission electron microscopy (TEM) and dynamic light scattering (DLS). (**a**,**b**) TEM-images of the prepared Au nanoparticles and size distributions according to (**c**) TEM particle analysis and (**d**) Zetasizer analysis.

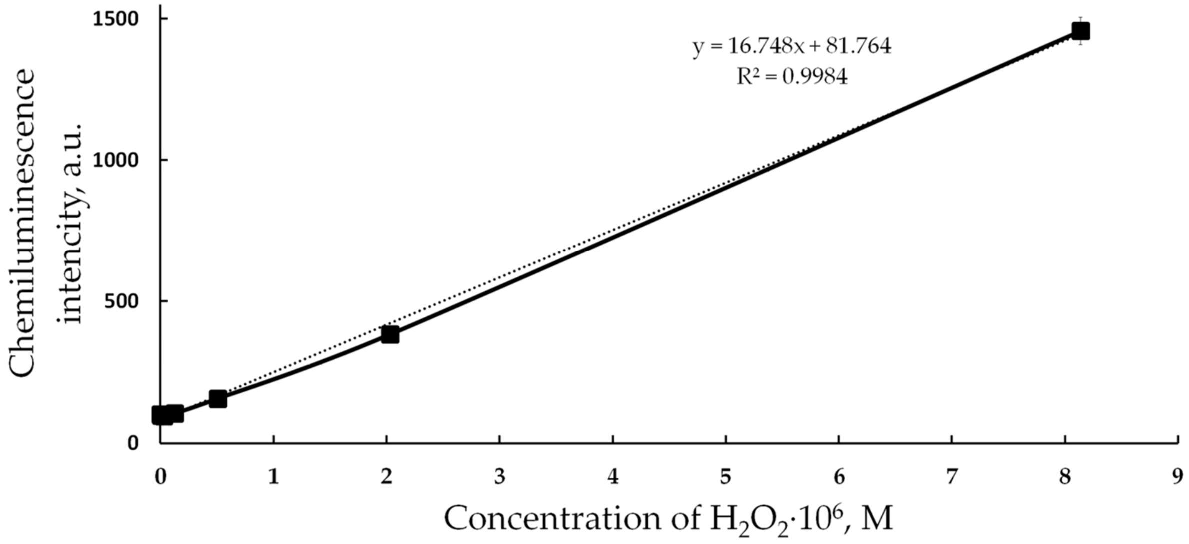

Figure S2. Calibration curve for chemiluminescence luminol method for ROS evaluation. Dependence of chemiluminescence intensity on concentration of H_2O_2. Dotted line: linear regression function. Solid line: experimental values.

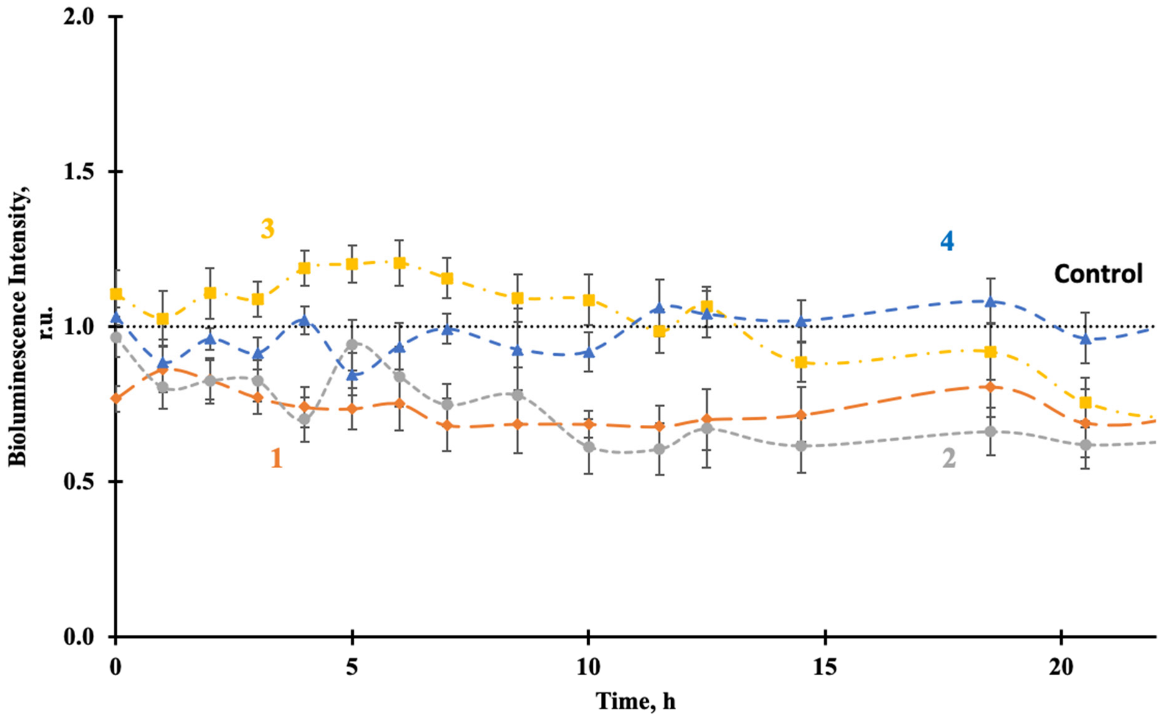

Figure S3. Bioluminescence kinetics of bacteria sampled at exponential phase of growth (17 h) in the presence of AuNPs. Concentrations of AuNPs were: (1) -10^{-3}, (2) -10^{-4}, (3) -10^{-5}, (4) -10^{-6} g/L.
